# Influence of *Heterodera glycines* Virulence Phenotypes on the Performance of Nematode-Protectant Seed Treatments

**DOI:** 10.2478/jofnem-2025-0032

**Published:** 2025-08-10

**Authors:** Jefferson Barizon, Kaitlyn Bissonnette, Meghan Biggs, Alexandria Haafke, Mandy Bish

**Affiliations:** Plant Sciences and Technology Division, University of Missouri, Columbia, MO 65211; Current Address: Agriculture and Environmental Research Division, Cotton Incorporated, Cary, NC 27513

**Keywords:** fluopyram, *Glycine max*, HG type, *Heterodera glycines*, ILEVO, phenotype, nematode-protectant seed treatment, soybean, soybean cyst nematode, virulence

## Abstract

Soybean cyst nematode (SCN; *Heterodera glycines*) is a major pathogen of soybean (*Glycine max*) in North America. Although nematodeprotectant seed treatments can complement SCN-resistant soybean varieties in managing this pathogen, their efficacy across different SCN virulence phenotypes remains unclear. This study evaluated whether SCN phenotype influences seed treatment performance and assessed treatment effects on SCN reproduction under controlled conditions. Seeds of the SCN-resistant variety P37A27X (PI 88788) were treated with a base fungicide and insecticide, or the base combined with Aveo EZ (*Bacillus amyloliquefaciens*); Bio ST (heat-killed *Burkholderia rinojensis*); Clariva PN (*Pasteuria nishizawae*); ILEVO (fluopyram); Saltro (pydiflumetofen); or Trunemco (cis-jasmone and *B. amyloliquefaciens*). Untreated resistant and susceptible seeds served as controls. One non-virulent (HG type 0) and two virulent (HG types 2.5.7 and 1.2.5.7) phenotypes were tested, and treatments were evaluated 30 days after planting. Base-and-ILEVO treatment reduced the population of SCN females by 29% to 72% across phenotypes, and also reduced root mass by 28%, indicating early phytotoxic effects.

Soybean (*Glycine max* [L.] Merrill) is one of the most economically important crops in the United States. In the 2024 growing season, 118.9 million metric tons (approximately 4.4 billion bushels) were harvested from 34.8 million ha (86 million acres), totaling $44.1 billion (NASS USDA, 2025). The soybean cyst nematode (SCN; *Heterodera glycines* Ichinohe) is considered the most problematic pathogen in North American soybean production. From 2015 to 2019, approximately 15.5 million metric tons (569.5 million bushels) of soybeans were lost due to SCN in the United States and Canada (Bradley et al., 2021).

Several factors contribute to the destructive potential of SCN. These include its broad geographical distribution (Tylka and Marett, 2021); its short life cycle and high reproduction rate, which enables rapid population increases (Lauritis et al., 1983; Niblack, 2006); and the ability to persist in soil for years in the absence of a host crop (Howard et al., 1998; Chen et al., 2001). In addition, increasing virulence against commercially available SCN-resistant varieties has compromised the long-term effectiveness of host resistance as a management strategy (Mitchum et al., 2007; Niblack et al., 2008; McCarville et al., 2017; Howland et al., 2018).

The increasing virulence of SCN field populations is largely attributed to overreliance on resistance genes derived from plant introduction PI 88788. These were present in approximately 90% of SCN-resistant soybean varieties included in Iowa variety trials for the 2024 season (Tylka and Mullaney, 2023), and this is likely representative of variety availability in other soybean-growing regions of the United States. In Missouri, surveys have documented an increase in the frequency of SCN detection in soybean fields, with recovery from 49% of samples in 2005, increasing to 88% in 2015 and 2016 (Mitchum et al., 2007; Howland et al., 2018). When these populations were tested for virulence on PI 88788, 78% of samples in 2005 had a female index (FI) > 10, indicating the ability to reproduce at more than 10% of the rate observed on a susceptible cultivar. By 2015 and 2016, 100% of SCN populations tested had an FI > 10, and 58% had an FI > 50 (Howland et al., 2018). Similar trends have been reported in Illinois, where Kleczewski et al. (2023) found that it was common for SCN populations to reproduce on PI 88788 soybean, with an average FI of 29.4.

Nematode-protectant seed treatments may enhance the performance of SCN-resistant soybean varieties by reducing early-season SCN reproduction. Several commercial seed treatments with biological or chemical active ingredients and distinct modes of action have been introduced over the past two decades (Davis and Tylka, 2021). Controlled environment studies have demonstrated their potential for influencing SCN hatching, mobility, mortality, root penetration, and reproduction (Beeman and Tylka, 2018; Jensen et al., 2018; Beeman et al., 2019). However, field trials of these treatments have shown inconsistent effects on SCN population densities and soybean yield (Gaspar et al., 2014, 2017; Kandel et al., 2017, 2018; Mourtzinis et al., 2017; Bissonnette et al., 2018; Wang et al., 2019; Dhital, 2020; Roth et al., 2020; Sjarpe et al., 2020; Kessler and Koehler, 2023).

To address the inconsistent performance of the nematode-protectant seed treatments observed in earlier field trials, Bissonnette et al. (2024) implemented a standardized protocol to evaluate six commercially available products for SCN management across multiple environments. These environments varied in the presence of sudden death syndrome (SDS; *Fusarium virguliforme*), which can reduce yield and tends to exhibit more prominent symptoms when SCN levels are high (Gao et al. 2006; Kandel et al. 2017), as well as in SCN population densities and virulence phenotypes. No treatment reduced the end-of-season SCN reproduction factor, regardless of initial SCN population density or SDS presence. However, ILEVO more significantly reduced the number of SCN females at 30 to 35 days after planting compared to Aveo EZ, Bio ST, Clariva, and Saltro, while showing similar effects to Trunemco and the controls.

A factor not examined in that study, but that may contribute to the variable responses of nematodeprotectant seed treatments, is SCN virulence phenotype. To complement the field-based evaluation, a controlled environment experiment was conducted to investigate how SCN virulence phenotypes influence the performance of nematode-protectant seed treatments.

## Materials and Methods

### SCN phenotypes

Three SCN populations representing HG types 0, 2.5.7, and 1.2.5.7 were selected from the University of Missouri Nematology Laboratory (SCN Diagnostics) collection. These HG types reflect the most frequently encountered virulence profiles across environments included in the Bissonnette et al. (2024) field trial, as well as in previous surveys of SCN populations in Missouri. An HG type test was conducted on each population prior to the greenhouse experiments to confirm their virulence profiles (Table 1; Niblack et al., 2002). SCN populations were maintained on a mixture of susceptible soybean cultivars (‘Williams 82’, ‘Essex’, and ‘Lee 74’), with cultures restarted every 60 days.

**Table 1:** SCN populations and virulence phenotypes (HG types).

**Soybean lines**	**HG Type 0**	**HG Type 2.5.7**	**HG Type 1.2.5.7**

	**Female Index (%)^a^**		
1. PI 548402 (Peking)	2.4	0.6	18.4
**2. PI 88788**	**5.8**	**86.1**	**92.7**
3. PI 90763	0.7	0.0	0.2
4. PI 209332	0.0	0.0	0.0
5. PI 209332	3.1	80.6	72.0
6. PI 89772	0.8	0.0	0.2
7. PI 548316 (Cloud)	9.5	76.0	74.0

a SCN Female Index = (Mean number of SCN females on PI / Mean number of SCN females on the SCN-susceptible Lee 74) × 100.

### Seed treatments and controls

Treatment selection was based on multi-state field trials conducted between 2019 and 2021 (Bissonnette et al., 2024); soybean seeds and seed treatments were sourced from the same lots used at the Missouri locations in that study. The SCN-resistant soybean variety P37A27X (relative maturity 3.7; PI 88788 source of resistance; Pioneer, Corteva Agriscience, Johnston, IA) was treated with fungicide-and-insecticide base containing fluxapyroxad, metalaxyl, pyraclostrobin, and clothianidin. This base was followed by one of six commercially available nematode-protectant seed treatments: Aveo EZ (*Bacillus amyloliquefaciens*); Bio ST (heat-killed *Burkholderia rinojensis*); Clariva PN (*Pasteuria nishizawae*); ILEVO (fluopyram); Saltro (pydiflumetofen); or Trunemco (cis-jasmone + *B. amyloliquefaciens*) (Table 2). The primary control was base-treated P37A27X. Two additional controls were included to confirm that SCN populations were reproducing as expected: untreated P37A27X and untreated SCN-susceptible ‘Williams 82’.

**Table 2:** Soybean cultivar P37A27X (PI 88788) and seed treatments used in greenhouse experiments conducted in 2020 and 2021, including active ingredients, application rates, and manufacturers.

**Treatment**	**Active ingredient(s)**	**Rate**	**Company**
Untreated	N/A	N/A	N/A
Base	metalaxyl, pyraclostrobin, fluxapyroxad, clothianidin	See footnote^a^	See footnote^b^
Base + Aveo EZ	*Bacillus amyloliquefaciens* PTA-4838	2 ml/100,000 seeds	Valent U.S.A. (San Ramon, CA)
Base + Bio ST	heat-killed *Burkholderia rinojensis* + fermentation media	195 ml/100 kg	Albaugh, LLC (Ankeny, IA)
Base + Clariva PN	*Pasteuria nishizawae*	130 ml/100 kg	Syngenta (Greensboro, NC)
Base + ILEVO	fluopyram	0.15 mg a.i. / seed	BASF (Research Triangle Park, NC)
Base + Saltro	pydiflumetofen	0.075 mg a.i. / seed	Syngenta (Greensboro, NC)
Base + Trunemco	Cis-jasmone, *Bacillus amyloliquefaciens*	20.2 ml / 100 kg	Nufarm (Alsip, IL)

a Base composition: metalaxyl (4 g a.i./100 kg), pyraclostrobin (7.5 g a.i./100 kg), fluxapyroxad (5 g a.i./100 kg), and clothianidin (0.11 mg a.i./seed).

b Bayer (Creve Coeur, MO) and BASF (Research Triangle Park, NC).

### Experimental setup

Four experimental replicates were conducted between 2020 and 2021, with two replicates performed each year. Each experiment included four blocks. On the day of experimental setup, inoculum from each SCN population was prepared by washing the soybean root systems to remove the soil, dislodging SCN females using a high-pressure stream of water, and collecting them on a 250 µm pore sieve. Harvested SCN females were crushed to release eggs (Faghihi and Ferris, 2000) and purified by sucrose centrifugation using a 454 g/L sucrose solution (Jenkins, 1964). Egg suspensions were adjusted to a concentration of 500 eggs per 1 mL of water.

For each inoculation, 1 mL of SCN egg suspension was pipetted into a 100 cm^3^ capacity PVC tube (3-cm diameter by 15-cm length) filled with steam-pasteurized sandy loam soil (72.5% sand, 17.5% silt, 10% clay; 0.5% organic matter; pH 7.4). The inoculum was placed approximately 5 cm below the soil surface. One soybean seed was placed in the center of the tube and pressed to a depth of 2 cm, then covered with the same soil. Twenty tubes were arranged in a plastic container (5.7-L capacity, 22.2-cm diameter, and 19.1-cm height; Parade Plastic, Mooresville, IN), and pasteurized sandy loam soil was used to fill the gaps between the tubes and hold them in place. Each container served as a block and included two replications per treatment, except for the untreated SCN-susceptible Williams 82, which was replicated four times per container. In total, eight plants were evaluated in each experiment for each combination of seed treatment and SCN phenotype.

Containers were suspended in a water bath maintained at 27 °C ± 1 °C under a 12-hour light-dark cycle in a randomized complete block design. Soybeans were watered daily and fertilized weekly with a water-soluble fertilizer (Tomato Plant Food; Miracle-Gro, Marysville, OH) according to the manufacturer’s instructions. At 30 days after planting, aboveground biomass was removed at the soil line and roots were gently washed to remove soil. SCN females were extracted using the same procedure as for inoculum preparation, transferred to a Petri dish, and counted under a dissecting microscope (SZX9; Olympus, Tokyo, Japan). Cleaned roots were placed in paper bags and dried at 70 °C for 48 hours, and root dry weights were recorded.

### Exclusion criteria

The SCN-susceptible soybean cultivar ‘Williams 82’ was included in each experiment as a biological check to validate nematode reproduction under experimental conditions. Data from ‘Williams 82’ were not included in the statistical analysis, but were used to determine whether SCN reproduction fell within an acceptable range for reliable treatment comparisons. Experimental replicates were excluded from the analysis if the average number of SCN females recovered from untreated ‘Williams 82’ roots was either < 100 or > 250. These thresholds reflect the expected range of SCN reproduction at the applied inoculum level under ideal greenhouse conditions. The lower bound of 100 SCN females represents the minimum acceptable level of reproduction (Niblack et al., 2009). The upper bound of 250 assumes that SCN males and females occur in approximately equal numbers, so that full establishment of the 500-egg inoculum would result in approximately 250 females (Koliopanos and Triantaphyllou, 1972).

For the non-virulent phenotype (HG type 0), data from the first experimental replicate of each year were excluded due to inconsistent SCN reproduction in the ‘Williams 82’ control. The average number of SCN females recovered from ‘Williams 82’ was 72.6 in 2020 and 296.6 in 2021, which fell outside the predefined bounds of < 100 or > 250. In the two experimental replicates retained for analysis, the average number of SCN females recovered from ‘Williams 82’ was 157 in 2020 and 145.7 in 2021, confirming acceptable reproduction. For all experimental replicates involving HG types 1.2.5.7 and 2.5.7, SCN reproduction in Williams 82 fell within the acceptable range, and no exclusions were necessary.

### Data Analyses

A Generalized Linear Mixed Model (GLMM) was implemented using the PROC GLIMMIX procedure in SAS (version 9.4, SAS Institute Inc., Cary, NC) to evaluate the effects of nematode protectant seed treatments and SCN phenotypes on three response variables (SCN females per root, SCN females per gram of dry root, and root dry weight). Seed treatment and SCN phenotype were specified as fixed effects, and experimental replicates were included as a random effect.

For SCN females per root and SCN females per gram of dry root, a GLMM with a negative binomial distribution and log link function was used. Each response variable was modeled as a function of seed treatment, SCN phenotype, and the interaction of the two. Differences among Least Squares Means (LSMEANS) were compared using Tukey’s adjustment for multiple comparisons, with significance set at α = 0.05. Results were presented on the inverse link scale.

Given the significant main effect of SCN phenotype, additional models were run for each phenotype with seed treatment as a fixed effect and experimental replicate as a random effect. Model fit statistics, including scale parameter estimates and covariance structure, were evaluated to account for variability and dispersion in each response variable. LSMEANS were compared using Tukey’s adjustment (α = 0.05).

Root dry weight was analyzed using a GLMM with a Gaussian distribution, with seed treatment, SCN phenotype, and the interaction of the two as fixed effects and experimental replicate as a random effect. Due to the non-significant effect of SCN phenotype, seed treatment means were pooled across phenotypes for comparison. All pairwise comparisons were conducted using Tukey’s adjustment (α = 0.05).

## Results

### SCN females per root

Seed treatment (F-value = 21.8, p < 0.0001) and SCN phenotype (F-value = 712.1, p < 0.0001) significantly affected the number of SCN females per root. However, their interaction was not significant (F-value = 0.9, p = 0.53).

The number of SCN females per root varied significantly by SCN phenotype. HG type 0 averaged 8.1 females (SE = 0.6), HG type 2.5.7 averaged 84.8 (SE = 4.5), and HG type 1.2.5.7 averaged 90.3 (SE = 4.9). No significant difference was observed between HG types 2.5.7 and 1.2.5.7 (estimate = 1.1, SE = 1.1, p = 0.33). However, HG type 2.5.7 produced 10.5 times more females per root than HG type 0 (SE = 1.1, p < 0.0001), and HG type 1.2.5.7 produced 11.2 times more (SE = 1.1, p < 0.0001).

Seed treatment affected the number of SCN females per root across all phenotypes (Table 3). The base-and-ILEVO treatment was the only one that resulted in significantly fewer SCN females per root compared to the base control. For HG type 0, the estimated number of SCN females was 2.6 (SE = 0.6) for base and ILEVO and 9.5 (SE = 1.8) for base alone. For HG type 2.5.7, estimates were 34.9 (SE = 4.1) for base and ILEVO and 103.4 (SE = 11.5) for base alone. For HG type 1.2.5.7, the estimated means were 41.5 (SE = 3.6) for base and ILEVO and 101.1 (SE = 8.2) for base alone. The other seed treatments applied with the base (Aveo EZ, Bio ST, Clariva PN, Saltro, and Trunemco) did not significantly differ from one another, or from the untreated resistant and base control.

**Table 3:** Number of soybean cyst nematode (SCN) females per root, 30 days after planting, for PI 88788 seed (variety P37A27X) treated with different seed treatments, by SCN phenotype.

**Treatment**	**HG type 0**			**HG type 2.5.7**			**HG type 1.2.5.7**		
		
	**Females per root^a^**	**SE**	**Group**	**Females per root**	**SE**	**Group**	**Females per root**	**SE**	**Group**
Untreated	9.0	1.7	A	106.9	11.7	A	119.2	8.8	A
Base	9.5	1.8	A	103.4	11.5	A	101.1	8.2	A
Base + Aveo EZ	11.7	2.1	A	90.7	10.4	A	90.6	7.0	A
Base + Bio ST	10.1	1.9	A	88.0	9.9	A	107.1	8.1	A
Base + Clariva PN	8.7	1.7	A	96.5	11.0	A	99.5	7.4	A
Base + ILEVO	2.6	0.6	B	34.8	4.1	B	41.5	3.6	B
Base + Saltro	9.9	1.8	A	91.4	9.9	A	94.9	6.9	A
Base + Trunemco	10.9	2.0	A	97.5	11.2	A	94.2	7.1	A
*Main effect*	*F-value = 6.5, p <.0001*			*F-value = 11.9, p <.0001*			*F-value = 14.7, p <.0001*		

a Means followed by the same letter are not significantly different based on the Least Square Means (LSMEANS, α = 0.05).

### SCN females per gram of dry root

Both seed treatment (F-value = 13, p < 0.0001) and SCN phenotype (F-value = 750.2, p < 0.0001) significantly affected the number of SCN females per gram of dry root. The interaction between the two was not significant (F-value = 1.5, p = 0.11).

The number of SCN females per gram of dry root also varied significantly by phenotype. HG type 0 averaged 38.7 females per gram of dry root (SE = 7.1); HG type 2.5.7 averaged 398.8 (SE = 70.1); and HG type 1.2.5.7 averaged 421.1 (SE = 74.6). No significant difference was found between HG types 2.5.7 and 1.2.5.7 (estimate = 1.1, SE = 1.1, p = 0.5), but both averaged more than 10 times higher than HG type 0 (10.3 × and 10.9 ×, respectively; both p < 0.0001).

Seed treatment affected the number of SCN females per gram of dry root across all SCN phenotypes (Table 4). The base and ILEVO combination was the only treatment that resulted in significantly lower numbers of SCN females compared to the base control. For HG type 0, the estimated number of SCN females per gram was 12.4 (SE = 2.2) for base and ILEVO and 34.3 (SE = 5.6) for base alone. For HG type 2.5.7, estimates were 243 (SE = 46.6) for base and ILEVO and 440 (SE = 83.2) for base alone. For HG type 1.2.5.7, estimates were 274.5 (SE = 57.1) for base and ILEVO and 487.9 (SE = 101.0) for base alone.

**Table 4:** Number of soybean cyst nematode (SCN) females per gram of root dry weight, 30 days after planting, for PI 88788 seed (variety P37A27X) treated with different seed treatments, by SCN phenotype.

**Treatment**	**HG type 0**			**HG type 2.5.7**			**HG type 1.2.5.7**		
		
	**Females per gram root^a^**	**SE**	**Group**	**Females per gram root**	**SE**	**Group**	**Females per gram root**	**SE**	**Group**
Untreated	33.7	5.6	A	486.1	91.4	A	508.8	103.5	A
Base	34.3	5.6	A	440.0	83.2	A	487.9	101.0	A
Base + Aveo EZ	43.7	6.9	A	451.1	86.5	A	394.6	80.8	AB
Base + Bio ST	37.4	6.0	A	401.5	76.3	A	467.9	95.5	A
Base + Clariva PN	31.1	5.1	A	442.9	84.7	A	441.5	89.9	A
Base + ILEVO	12.3	2.2	B	243.0	46.6	B	274.5	57.1	B
Base + Saltro	39.7	6.2	A	400.9	75.1	A	454.7	92.3	A
Base + Trunemco	42.2	6.9	A	393.9	75.3	A	394.4	80.6	AB
*Main effect*	*F-value = 6.6, p < .0001*			*F-value = 3.9, p = .0005*			*F-value = 4.8, p < .0001*		

a Means followed by the same letter are not significantly different based on the Least Square Means (LSMEANS, α = 0.05).

For HG type 1.2.5.7, base-and-Aveo EZ and base-and-Trunemco treatments resulted in similar SCN females per gram of dry root as the base-and-ILEVO treatment; however, they also did not differ from the base control or any other treatment. The other seed treatments (base with Bio ST, Clariva PN, and Saltro) did not significantly differ from one another, or from the base or untreated resistant control.

### Root dry weight

Seed treatment significantly affected soybean root dry weight (F-value = 14.3, p < 0.0001), but the effects of SCN phenotype (F-value = 2.7, p = 0.07) and seed treatment by phenotype interaction (F-value = 1.0, p = 0.49) were not significant. Because SCN phenotypes were not significant, they were combined for analysis of seed treatment effects (Table 5). The estimated root dry weight for the base-and-ILEVO treatment was 0.17 g (SE = 0.03) — significantly lower than the base control, which had an estimated weight of 0.24 g (SE = 0.03). All other seed treatments (base combined with Aveo EZ, Bio ST, Clariva PN, Saltro, or Trunemco) did not significantly differ from one another, or from the base or untreated resistant control.

**Table 5:** Soybean root dry weight, 30 days after planting, across all SCN phenotypes for PI 88788 seed (variety P37A27X), treated with different seed treatments.

**Treatment**	**Root dry weight (g)^a^**	**SE**	**Group**
Untreated	0.24	0.03	A
Base	0.24	0.03	A
Base + Aveo EZ	0.24	0.03	A
Base + Bio ST	0.25	0.03	A
Base + Clariva PN	0.24	0.03	A
Base + ILEVO	0.17	0.03	B
Base + Saltro	0.23	0.03	A
Base + Trunemco	0.25	0.03	A
*Main effect*	*F-value = 14.3, p < .0001*		

a Means followed by the same letter are not significantly different based on the Least Square Means (LSMEANS, α = 0.05).

## Discussion

This study evaluated whether SCN virulence phenotype affected the performance of six commercially available nematode protectant seed treatments under controlled conditions. The tested phenotypes were HG types 0, 2.5.7, and 1.2.5.7, which differ in their ability to reproduce on PI 88788, the most widely deployed source of SCN resistance in commercial soybean varieties. Reproduction varied significantly among phenotypes, with HG types 2.5.7 and 1.2.5.7 producing substantially more females than HG type 0. These results aligned with HG type tests conducted prior to the experimental setup, which showed female indices on PI 88788 of 5.8 for HG type 0, 86.1 for HG type 2.5.7, and 92.7 for HG type 1.2.5.7. These findings further highlight the erosion of the resistance conferred by PI 88788.

Although SCN phenotype influenced the magnitude of reproduction, there was no significant interaction between phenotype and seed treatment, indicating that treatment effects were consistent across HG types. Therefore, SCN virulence phenotypes may not be a primary factor in the variability in seed treatment efficacy across environments. The base-and-ILEVO treatment was the only one that significantly reduced the number of SCN females per root and SCN females per gram of dry root compared to the base control. Reductions in females per root were 72% for HG type 0; 66% for HG type 2.5.7; and 59% for HG type 1.2.5.7. When adjusted for root dry weight, reductions were 64%, 29%, and 44%, respectively.

Field trials with ILEVO have been inconsistent, but there have been observations of ILEVO reducing the number of SCN females per gram of dry root in some environments (Kessler and Koehler, 2023; Bissonnette et al., 2024). In a field study (Kessler and Koehler, 2023), ILEVO reduced the number of females per gram of root compared to non-treated seed and Saltro at some, but not all, locations. In another field study by Bissonnette et al. (2024), base-and-ILEVO treatment reduced the number of SCN females per root and per gram of root at 30 to 35 days after planting compared to several other treatments. However, the results did not differ from base-and-Trunemco treatment, untreated, and the base control. These effects were primarily observed in a single-environment grouping characterized by high initial SCN density and low SDS pressure.

Base-and-ILEVO treatment also significantly reduced root biomass. Mean root dry weight in treated plants was 0.17 g, compared to approximately 0.24 g in the base control —a 28% reduction. Fluopyram, the active ingredient in ILEVO, is a succinate dehydrogenase inhibitor (SDHI) that may impair early root development by inhibiting mitochondrial respiration (Rocha et al., 2022). Although yield suppression from ILEVO has not been consistently reported (Kandel et al., 2018), reduced root mass could compromise plant resilience under environmental stress.

The decreased SCN reproduction observed with base-and-ILEVO treatment may be partially influenced by lower root biomass, which would reduce the number of available feeding sites. However, consistent reductions in both the number of total females and the number of females per gram of root suggest a direct nematicidal effect. Prior studies support this interpretation. Beeman et al. (2019) found that fluopyram suppressed SCN penetration at 2.5 cm but not at greater depths, indicating limited mobility or residual activity. Similarly, Hawk and Faske (2020) reported that seed-applied fluopyram suppressed *Meloidogyne incognita* root penetration up to 5 cm in cotton and up to 2.5 cm in soybean. These findings suggest that fluopyram may act directly on nematodes near the root zone, especially during early infection stages.

In controlled conditions, where the seed, treatment, and inoculum were confined within narrow plastic tubes, SCN eggs and juveniles are likely to have more exposure to the active ingredients. Unlike in field settings, where soybean roots can grow laterally and away from the treated seed zone, the 3-cm diameter-by-15-cm-length tubes limited lateral root expansion and directed growth downward, causing roots to curl and remain near the treated seed. This vertical orientation, combined with frequent watering, may have mobilized the fluopyram along the root path, increasing its contact with the nematodes. This setup thus likely enhanced the nematicidal activity of the fluopyram.

This study demonstrated that SCN virulence phenotypes are not a primary factor in determining the efficacy of nematode-protectant seed treatments. Base-and-ILEVO treatment provided the most consistent reductions across all SCN phenotypes in a controlled environment. However, seed treatments can only partially mitigate losses caused by increasingly virulent SCN populations. The SCN virulence phenotypes tested in this study (HG types 2.5.7 and 1.2.5.7) had higher female SCN counts compared to the non-virulent population (HG type 0), even when resistant soybean was treated with ILEVO.

These results highlight both the potential and the limitations of seed-applied treatments and underscore the need for integrated SCN management strategies that include resistance diversification, crop rotation, and routine monitoring of SCN. Continued development of more effective seed treatments and novel resistance sources will be critical for long-term SCN management.
